# Building COPD care on shaky ground: a mixed methods study from Swedish primary care professional perspective

**DOI:** 10.1186/s12913-017-2393-y

**Published:** 2017-07-10

**Authors:** Sara Lundell, Malin Tistad, Börje Rehn, Maria Wiklund, Åsa Holmner, Karin Wadell

**Affiliations:** 1 Department of Community Medicine and Rehabilitation, Physiotherapy, Umeå University, 901 87 Umeå, Sweden; 2 School of Education, Health and Social Studies, Dalarna University, 791 88 Falun, Sweden; 3 Department of Radiation Sciences, Umeå University, 901 87 Umeå, Sweden

**Keywords:** Chronic obstructive pulmonary disease, Primary care, Healthcare professionals, Mixed methods, Healthcare system, Organisation, Implementation, Health promotion, Treatment guidelines, Sweden

## Abstract

**Background:**

Chronic obstructive pulmonary disease (COPD) is a public health problem. Interprofessional collaboration and health promotion interventions such as exercise training, education, and behaviour change are cost effective, have a good effect on health status, and are recommended in COPD treatment guidelines. There is a gap between the guidelines and the healthcare available to people with COPD.

The aim of this study was to increase the understanding of what shapes the provision of primary care services to people with COPD and what healthcare is offered to them from the perspective of healthcare professionals and managers.

**Methods:**

The study was conducted in primary care in a Swedish county council during January to June 2015. A qualitatively driven mixed methods design was applied. Qualitative and quantitative findings were merged into a joint analysis. Interviews for the qualitative component were performed with healthcare professionals (*n* = 14) from two primary care centres and analysed with qualitative content analysis. Two questionnaires were used for the quantitative component; one was answered by senior managers or COPD nurses at primary care centres (*n* = 26) in the county council and the other was answered by healthcare professionals (*n* = 18) at two primary care centres. The questionnaire data were analysed with descriptive statistics.

**Results:**

The analysis gave rise to the overarching theme *building COPD care on shaky ground*. This represents professionals driven to build a supportive COPD care on ‘shaky’ organisational ground in a fragmented and non-compliant healthcare organisation. The shaky ground is further represented by uninformed patients with a complex disease, which is surrounded with shame. The professionals are autonomous and pragmatic, used to taking responsibility for their work, and with limited involvement of the management. They wish to provide high quality COPD care with interprofessional collaboration, but they lack competence and are hindered by inadequate routines and lack of resources.

**Conclusions:**

There is a gap between COPD treatment guidelines and the healthcare that is provided in primary care. To facilitate implementation of the guidelines several actions are needed, such as further training for professionals, additional resources, and improved organisational structure for interprofessional collaboration and patient education.

**Electronic supplementary material:**

The online version of this article (doi:10.1186/s12913-017-2393-y) contains supplementary material, which is available to authorized users.

## Background

Chronic obstructive pulmonary disease (COPD) is a major public health problem, an economic burden and the cost of healthcare is directly related to the severity of the disease [1, 2]. About 65 million people worldwide are estimated to have moderate to severe COPD [3], but the disease is largely underdiagnosed, and the prevalence is consequently difficult to estimate [4, 5]. Therefore, provision of diagnostics by the healthcare system is important. Chronic airflow limitation caused by an inflammation in the smaller airways characterises COPD and the major symptom is dyspnoea [1]. Weight loss, muscle wasting, decreased physical capacity, heart failure, osteoporosis and depression are also common in people with COPD, all leading to lower quality of life [1, 6, 7]. Furthermore, the level of physical activity is markedly reduced [8–10]. This is alarming since level of physical activity is one of the strongest predictor of all-cause mortality in COPD [11].

Pulmonary rehabilitation (PR) is an important treatment and health promotion intervention that should be provided to people with COPD. PR consists of patient-tailored therapies that include, but are not limited to, exercise training, education, and behaviour change (e.g. increasing physical activity level). PR is cost-effective and has positive effects on quality of life, dyspnoea, anxiety, depression, and physical capacity [12–17]. Access to hospital-based PR is <0.5% in Sweden [18], <1% in the UK [19], and 1.2% in Canada [20]. In addition, a third of the people with COPD that were offered hospital-based PR in Sweden declined participation [18].

Whether people with COPD are treated in hospitals or in primary care varies internationally [21, 22]. In Sweden, most were previously treated in hospital specialty clinics, but now most are treated in primary care. Recent studies [23, 24] demonstrated that the availability of healthcare professionals for rehabilitation is high in primary care. Nevertheless, people with COPD had no access to PR in a quarter of the primary care centres [23]. There is an obvious gap between the recommended provision of PR and the actual availability for people with COPD.

The framework integrated-Promoting Action on Research Implementation of Health Services (i-PARIHS) points to the interplay of factors during the complex process of implementation of research evidence into clinical practice. The innovation, the recipients, the context (including management, organisation and culture), and how implementation of the innovation is facilitated are important components for successful implementation [25]. eHealth has been suggested as a promising model when aiming to enhance implementation, use, and delivery of pulmonary rehabilitation [12, 26]. However, implementation of a new practice requires knowledge about the present primary care situation with regard to the delivery of healthcare to people with COPD. In order to provide foundation for a project aiming at developing an eHealth tool to support primary care health promotion interventions for people with COPD, we designed an exploratory study to map out COPD care in primary care.

### Aim

The aim of the present study was to increase the understanding of what shapes the provision of healthcare services to people with COPD and what healthcare is offered to people with COPD, from the perspective of healthcare professionals and managers in primary care.

## Methods

The study was conducted in primary care in the Västerbotten County in Northern Sweden, which in 2016 had a population of approximately 266,000 [27]. Almost 80% of the population live in cities in the coastal area, while the remaining 20% live in large, sparsely populated areas interconnected by small municipalities in the inland. Sweden has among the oldest population in the world [28] and in Västerbotten County the proportion of people older than 80 years is slightly higher than the national average (about 5%). In fact, in some of the more sparsely populated municipalities in the inland every tenth person is above this age [27]. Healthcare services in Sweden are publicly funded and organized by 20 autonomous county councils and regions. Since 2010, all citizens have the right to enrol with any public or private primary care centre of their choice. The primary care centres consist of outpatient facilities. However, Västerbotten County Council, together with a few other county councils in the northern region, have some community hospitals in the inland. These community hospitals are organised under primary care, but also provides emergency care and are equipped with inpatient facilities along with other extended services, such as x-ray imaging.

### Study design

**Fig. 1:**
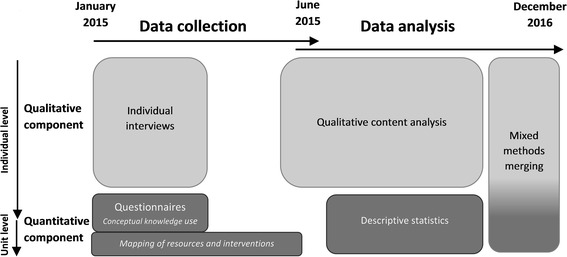
Timeline for the data collection and data analysis in the mixed methods design

A convergent, parallel mixed methods design [29–31] was used. Qualitative and quantitative data were collected and analysed in parallel, but separately. In the qualitative component, interviews were undertaken with healthcare professionals to explore their perception of what shapes the healthcare provided to people with COPD. In the quantitative component, two questionnaires were used to assess healthcare provided to people with COPD, as well as contextual factors and attitudes that might guide the provision of services. The final integration of data was performed by merging the data using a weaving approach [30] (Fig. 1). The mixed methods design was qualitatively driven; the qualitative component was considered the main component and the quantitative component was used to give an additional dimension to the results [32].

### Qualitative component

For the qualitative component two primary care centres were chosen. One centre is in a city with 120,000 inhabitants, and one is in a rural, sparsely populated area with 2500 inhabitants. In addition to covering both rural and urban settings, the centres were chosen based on the availability of designated nurses responsible for COPD care (henceforth referred to as “COPD nurses”). The urban centre had 7500 citizens enrolled. The rural centre had 2500 citizens enrolled. One of the primary care centres is private and the other public, although both are publicly funded and obligated to provide the same services.

The COPD nurses supported the researchers by identifying participants for recruitment. A maximum variation sample was striven for with regard to sex/gender and profession. Seventeen professionals were invited to participate in interviews. The number of participants was partly restricted due to the number of available professionals at the centres.

### Data collection and analysis

Qualitative data collection lasted from January 2015 to March 2015 (Fig. 1). Semi-structured face-to-face interviews with open-ended questions were performed by the second author (MT). The interview guide (Additional file 1) was inspired by the PARIHS framework [33] and factors known to be important for implementation of clinical guidelines [34, 35]. It covered questions about the professionals’ experience and perception of working with patients with COPD, and what influences the healthcare they provide to patients with COPD. Questions also covered how they work with routines and guidelines and how they follow up their work. All of the interviews started with the opening question “What is your experience of care and treatment for people with COPD?” The interviews lasted between 30 and 60 min. They took place at the primary care centre or a restaurant, based on the participants’ wishes. Interviews were audio recorded and transcribed verbatim by a professional transcriber. Three professionals (two medical social workers and one occupational therapist) declined participation because of limited time at the centre, lack of experience with COPD, or illness. Nine women and five men with different professions (district nurses/COPD nurses, physicians, frontline and senior managers, physiotherapists, dieticians and occupational therapists) were interviewed. Mean age was 47 (SD 9.5) years and they had a mean professional experience of 19 (SD 10) years.

Data from the interviews were analysed inductively using qualitative content analysis as described by Graneheim and Lundman [36]. The first author (SL) had primary responsibility for conducting the analysis in close collaboration with the second author (MT). First the interviews were read several times to gain a sense of the meaning as a whole. Then the interviews were divided into meaning units containing text that covered similar content. The meaning units were condensed and labelled with a descriptive code close to the manifest content. OpenCode 4.02 [37] was used during the coding procedure. Thereafter, the codes were compared based on similarities and differences, and grouped into subcategories and categories that described the manifest content of the interviews [36]. Based on the latent meaning, a theme was formulated [36]. As part of the triangulation between researchers [36], one additional researcher (MW) read and analysed a selected sample of interviews, and took part in the coding, categorising, and analytical abstraction. During the entire process, the analysis moved back and forth between the whole and parts of the text [36], and the emerging analysis was repeatedly discussed and compared between the three analysing authors (SL, MT and MW). Each author contributed with different perspectives and competences. The qualitative component was reported as recommended in standards for reporting qualitative research [38].

### Quantitative component

Two different samples were chosen for the quantitative component. The first sample consisted of the senior managers at all primary care centres (*n* = 39), henceforth referred to as “centres”, in the Västerbotten County Council in Sweden. The second sample consisted of all professionals who could potentially meet patients with COPD in their clinical work at the two centres included in the qualitative component (*n* = 22). To be able to compare some of the data with the qualitative component there was an overlap with the qualitative sample, as suggested by Creswell and Plano Clark [29].

### Data collection and analysis

The quantitative data collection lasted from January 2015 to June 2015 (Fig. 1). Two questionnaires were constructed specifically for this study and tested for face validity in specialty care and primary care contexts prior to the study.

*Mapping of resources and interventions* (Additional file 2) was based on a list of criteria for approved or optimal asthma/COPD reception in primary care [39] and evidence-based national guidelines for COPD [40]. The questionnaire addressed questions about which professionals are working with people with COPD, further training of the professionals, compliance with guidelines, availability of equipment and interventions, as well as difficulties related to health promotion interventions for people with COPD. The questionnaire was administered as a web survey to the first sample (senior managers). Reminders were sent to non-responding senior managers, to front-line managers and COPD nurses at the centres. Some centres were reminded by telephone. Answers were returned from 26 of 39 (67%) centres. They had a median of 5808 enrolled citizens (range 1050–18,800 citizens). A COPD diagnosis was documented in a median of 0.65% of the enrolled citizens (range 0–2.6%).

C*onceptual knowledge use* (Additional file 3) refers to knowledge that influences how staff think about specific issues, e.g., their attitudes and intentions [41]. The questionnaire addressed questions regarding professional attitudes, knowledge, and self-experience of readiness in delivering health promotion interventions to people with COPD. The questionnaire was administered to the second sample (professionals at two centres). One reminder was sent after a few weeks. Answers were returned from 18 of 22 (82%) professionals; 12 women and six men with different professions (district nurses/COPD nurses, physicians, physiotherapists and occupational therapists). Mean age was 49 (SD 12) years and mean professional experience was 21 (SD 10) years. Respondents had worked in their present position for a median of 4.5 (range 1–34) years.

Descriptive analyses were performed by one of the authors (BR) using IBM SPSS Statistics, version 23. Frequency tables were used, showing number of answers, central values, and allocation for each answer category. Means and standard deviations were calculated for normally distributed variables. Median and ranges were calculated for non-normally distributed variables.

### Mixed methods merging

When the analyses for the qualitative and quantitative components were completed, the responsible researcher for each component (SL and BR) discussed how to merge the findings. SL prepared a presentation of the results according to the agreement, and the merging was discussed and adjusted until satisfaction of both researchers. The results were then presented to all the authors, and the merging was discussed and adjusted.

This study is reported as recommended for mixed methods studies [42]. The results are presented with the qualitative component as the main story, and the quantitative complemental component [32] is merged with a weaving approach on a category-by-category basis [30]. In order to enhance the qualitatively driven design [32], the quantitative findings are presented descriptively in the running text with references to tables.

## Results

A theme, *Building COPD care on shaky ground,* that runs through the categories and subcategories was formulated during the qualitative analysis and is supported by the quantitative findings. The four categories, *The (un)demanding patient group*, *The non-compliant healthcare organisation*, *The challenged professionalism* and *The autonomous staff,* are built from 13 subcategories (Table 1). The theme, categories, and subcategories are presented below together with quotes from participants. The results from the questionnaires are interwoven into the main story and referred to in Tables 2 or 3.

**Table 1:** Theme, categories and subcategories in the qualitative component

*Theme*	Building COPD care on shaky ground			
*Category*	The (un)demanding patient group	The non-compliant healthcare organisation	The challenged professionalism	The autonomous staff
*Sub-category*	Shameful and low status disease	Fragmented healthcare	Demanding health promotion	Individual and shared responsibility
	Limited insight	Lacking competence and resources	Driven by a desire to do good	Resistance to top-down directives and hierarchies
	Grateful for little	Inhibited interprofessional collaboration	Tailoring interventions instinctively	Systematic versus pragmatic care development
				Driving spirit carries COPD care

**Table 2:** Answers (*n* = 26) from the questionnaire “Mapping of resources and interventions”

Measure	Centres
*Staffing*	
Nurse with responsibility for COPD and asthma	85%
Physician with responsibility for COPD and asthma	58%
Physiotherapist	61%
Assistant nurse	42%
Dietitian	35%
Occupational therapist	31%
Medical social worker	19%
Psychologist	19%
*Further training and time for COPD*	
Further training for COPD care	77%
Nurse with University credits within COPD and asthma (7,5 credits or more)	31%
Nurse, hours per week spent for working with:	
- patients with COPD, median (range) - patients with COPD and asthma, median (range)	4 (0–15) 8 (2–30)
*Equipment*	
Spirometer	100%
Pulse oximeter	100%
Oxygen	96%
Nebuliser	84%
*Examinations and interventions*	
Spirometry	88%
Spirometry and reversibility test	85%
Treatment of exacerbations	85%
Follow up of prioritised patients	58%
Care programme for COPD care (National or Regional)	77%
Routinely offers health promotion for COPD	38%
Smoking cessation support	85%
Assessment of symptoms with CAT	38%
Assessment of symptoms with mMRC	19%
6-min walk test	23%
Patient education in groups (with several professions involved)	15%
Written treatment plan	15%
*Difficulties with health promotion for COPD*	
Lack of resources	38%
COPD patients not interested	27%
Travel problems for COPD patients	23%
Not a prioritised group	8%
Lack of adequate knowledge among professionals	8%
Professionals not convinced about effects	0%

*Abbreviations*: *CAT* Chronic obstructive pulmonary disease Assessment Test, *COPD* Chronic obstructive pulmonary disease, *mMRC* modified Medical Research Council scale

Primary care centres in Västerbotten County Council in Sweden. Percentages (%) of primary care centres are reported

**Table 3:** Answers (*n* = 18) from the questionnaire “Conceptual knowledge use”

	It is important for primary care to offer …….. for people with COPD				Do you think that you have sufficient knowledge to give …….. to people with COPD?		Is it part of your work to give …….. to people with COPD?
*Category*	Don’t Agree	Agree Somewhat	Mostly Agree	Totally Agree	No, I don’t have sufficient knowledge	Yes, I have sufficient knowledge	Yes
*… health promotion interventions…*	0	1	4	13	11	7	10
*…tobacco prevention…*	0	0	3	15	7	11	8
*… disease-specific education …*	0	2	7	9	13	5	6
*…information about COPD-related self-care…*	1	0	3	14	9	9	9
*… consultative discussions about physical activity/exercise training…*	0	1	7	10	13	5	9
*…instructions and training in breathing techniques…*	0	3	5	10	14	4	4
*…advisory conversation about nutrition and energy needs…*	0	5	3	10	14	4	4
*…information about energy conservation techniques and assistive devices…*	0	5	3	10	14	4	3

### Building COPD care on shaky ground

The overarching theme captures driven and autonomous healthcare professionals who seek to build supportive and empowering COPD care on ‘shaky ground’, which is the fragmented and non-compliant healthcare organisation. The theme also captures perceptions of COPD as a complex condition permeated with shame and with low status in the healthcare organisation. The ‘shakiness’ is further reinforced by perceptions of the patients with COPD as a relatively uninformed group, who are indebted for the healthcare they receive. On this ‘shaky ground’, professionals are understood to be dedicated and pragmatic, and used to controlling their work with little involvement from the management. In a healthcare organisation, seen as simultaneously flat and somewhat hierarchal, the professionals wish to provide high quality COPD care based on interprofessional collaboration – but they lack competence and are hindered by obstacles they have to overcome, such as inadequate routines and lack of resources.

### The (un)demanding patient group

This category represents the professionals’ view of the group of patients with COPD as both undemanding and demanding. The patients were indebted for the healthcare they received, and this might be a consequence of the shame that surrounds their low status disease, along with their lack of knowledge about COPD. This results in a demanding situation for the professionals.

### Shameful and low status disease

COPD was perceived to be associated with shame, and the professionals had to be aware of this in their work. For example, many patients blamed themselves for causing their disease by smoking, and did not want to acknowledge it:

*COPD can involve a sense of guilt, since it has so much to do with smoking /…/. The persons brought it on themselves… (Professional 1)*

Low status and priority of COPD in the healthcare organisation was perceived as another demand. COPD was treated as less important and less interesting than other chronic conditions: “It’s sort of not as ‘special’”. Patients with COPD were prioritised lower than patients with diabetes for regular follow-ups, quality registers, and advice on physical activity. Only two centres reported COPD as low priority (Table 2). At the same time, only one third of the COPD nurses had undergone supplementary training about COPD and asthma at the university level, and they used 4 h per week (40 min/week per 1000 enrolled citizens) for patients with COPD (Table 2). A frustrated view was that management prioritised time for professional diabetes supplementary training, but no such time was given to professional COPD supplementary training. Instead, an investment in time for the COPD nurses was seen as a wise use of resources:

*[It’s important] to understand that having an asthma/COPD nurse is an investment that pays for itself right away. /…/ Primary care centres /…/ should be able to see for themselves what a relief it is for other staff, and that the result is a better operation. (Professional 2)*

### Limited insight

Another demanding part that contributed to the ‘shaky ground’ was insufficient patient insight and knowledge about COPD, its severity and its symptoms. One third of the centres stated that patients were not interested in participating in health promotion interventions (Table 2). According to the professionals, sometimes patients were not even aware that they suffered from COPD, and instead believed that they had asthma. Although some professionals viewed patients as knowledgeable enough, the dominant view was that many patients failed to understand the significance of seeking timely healthcare when symptoms were worsening, and an exacerbation was coming.

*They’ve become so ill by the time they make the call, that you can hear what a hard time [they’re having]… They’ve already been struggling for quite a while. (Professional 3)*

### Grateful for little

Patients with COPD were considered a quiet and undemanding group that rarely expressed requests or opinions and therefore had very low impact on the healthcare offered. However, the professionals said that patients seemed to appreciate the interventions they received and were positive when they realised they could help themselves by self-care.

*But I mean these aren’t demanding patients who’ve read extensively about the subject. They might occasionally mention* ‘I read about this in the newspaper.’ *These patients tend to be unassuming types who are happy with what they get. (Professional 2)*

### The non-compliant healthcare organisation

This category highlights a shaky organisational base represented by a healthcare system with divided responsibilities. This bothered the professionals, along with lack of competence, time, staffing, and collaboration that limited their work with COPD.

### Fragmented healthcare

The responsibility for the healthcare for patients with COPD was divided between levels in the healthcare organisation. Primary care handled most of the healthcare and rehabilitation, but patients with extensive needs could be referred to hospital-based specialty care for education and rehabilitation. In parallel, the municipality had responsibility for assistive technology and home adaptations. General dentistry sometimes handled support for smoking cessation. At times, this was thought to be confusing and unclear in terms of responsibility:

*“…and there are unbelievably poor guidelines as to what’s supposed to be primary care and what’s supposed to be specialty care. /…/ Working in primary care is actually utterly impossible now, since our responsibilities are so blurred. (Professional 4)*

### Lacking competence and resources

Lack of competence and resources in primary care was another perceived limitation. In interviews and questionnaires the professionals reported lack of knowledge, except for preventive work for tobacco use (Table 3). Some expressed feelings of insecurity due to no or very limited experience of working with patients with COPD. Keeping updated on the wide range of diseases and conditions covered by primary care was perceived to be difficult and they requested more knowledge. In contrast, only a few centres reported lack of knowledge as a difficulty for offering health promotion (Table 2). A majority of the centres reported that they had further training about COPD (Table 2), while the professionals explained that further training mainly came from pharmaceutical companies. The opportunities for other further training was seen as limited, and individuals were responsible for their own professional development.

*… I wanted to take some courses, but you’re not allowed to do that. You have to take some time off and do it on your own time, or use annual leave days for it, or something like that. (Professional 5)*

More than a third of the centres reported a lack of resources as a difficulty with providing COPD care (Table 2). Some upset professionals described how primary care was “constantly burdened with new assignments, without prior discussion or provision of additional resources”. They felt worried, pressured, and frustrated by this huge mission. Primary care was described as more dependent than specialty care on allocated resources and economic incentives to maintain service levels, which was pointed out as unfair. Limited availability of professionals and difficulty in recruiting professionals to rural areas were also seen as limiting their work with patients with COPD. To compensate for lacking resources they sometimes had to down-prioritise important tasks such as support for tobacco cessation or advice on physical activity. In order to catch up, they worked at a high speed with what they defined as “quick fixes”.

*So, we’re trying to pull things out of thin air every day to make this operation work, in terms of patients’ needs and what we can do… (Professional 4)*

Rather than working in this manner they wanted the management to provide enough time and to understand the importance of their work.

### Inhibited interprofessional collaboration

The professionals wanted to work with interprofessional collaboration, but they were limited by the organisation and current working methods. Interprofessional collaboration was thought to provide better knowledge and overview, to give insight into what the other professions do, and afford an opportunity to do more prevention work. Only a few centres reported that they offered patient education involving different professionals (Table 2). Most of the centres reported that they had a COPD nurse, and about half the centres had physiotherapists working with COPD care and a designated physician (Table 2). Other professions were less involved (Table 2), and about one third of the professionals did not encounter any patients with COPD during an ordinary week (Table 3). Further, the professionals pointed out that in small centres opportunities for interprofessional collaboration were limited or impossible:

*The medical social worker comes one day a week, or every other week or something, and the occupational therapist [comes] once every three weeks, or something like that. So, you can’t really have a team, because then, for that person, too much time would be lost doing other things. (Professional 5)*

Insufficient knowledge, along with some scepticism about other professions’ working methods were mentioned and potentially inhibited collaboration. This since establishing teams in different areas was their own responsibility. Some professions, such as physiotherapists, did not have clear roles in the existing teams. Instead, the core of the team (physicians and nurses) chose which patients other professions should meet.

#### The challenged professionalism

This category captures how the healthcare professionals wanted to be proud of their work and with an instinctive feel give their patients the best tailored care possible, including health promotion interventions. However, their professionalism was challenged by insufficient competence, routines and varying external conditions.

### Demanding health promotion

According to both interviews and questionnaires, an essential but demanding part of COPD care was health promotion focused on lifestyle changes, such as advice about physical activity and tobacco cessation (Table 3). Less than half of the centres routinely offered health promotion interventions for COPD, while most of the centres offered support for tobacco cessation (Table 2). All centres reported that their professionals were convinced about the effects of health promotion in COPD (Table 2). Only half of the professionals reported that health promotion was part of their work tasks (Table 3). They met many of the patients only during an acute exacerbation, which was not perceived as the right moment to start talking about exercise and lifestyle changes. To prevent rapid worsening, such as weight loss, inactivity and exacerbations, the patients should obtain a lifestyle change early in the disease. However, the general societal perception of weight loss as something positive and desired, could hinder the early discovery of unintended weight loss among patients with COPD.

Lack of time, unfamiliarity, lack of knowledge and comorbidities were mentioned as challenges for health promotion for COPD, along with getting together enough patients and finding suitable premises for group activities, and access to organised physical activities. Unequal requisites, such as patients’ poor economy and travel issues for the patients were also challenges mentioned in the interviews, and the later also in questionnaires (Table 2), while support from family members was perceived to have a positive impact.

### Driven by a desire to do good

Despite given challenges, professionals were driven by a desire to do good for their patients. Overall, working with patients with COPD gave meaning and joy, expressed as: “they’re among my best patients”. Developing a “primary care relationship” with the patients was viewed as important; meaning that the professionals got to know and developed a more holistic view when meeting the patients regularly and over time, “not just like ‘in and out’”. This gave health promotion a more natural part in the conversation and made the visits more efficient. It was also perceived as important to be able to be proud of one’s work and to know that the patients got the relevant interventions and equal healthcare:

*And consistent – that we’ll have discussed everything with each other so thoroughly that we won’t have the situation where someone can have good luck and call a nice nurse and everything goes well, or bad luck, call a nasty nurse, and things go badly. No, we need to have the same boundaries and guidelines. (Professional 6)*

### Tailoring interventions instinctively

Being flexible and tailoring interventions to the patients’ various needs, along with capturing patients with COPD early in their disease was seen as important. Spirometry tests was described as prioritised, which was confirmed in questionnaires since all centres were equipped with a spirometer and most centres offered spirometry with or without a reversibility test (Table 2).

When the diagnosis was set, professionals stated that the patients should be involved in their own healthcare. A majority thought it was important to offer disease-specific education (Table 3). The professionals wanted to educate the patients and provide them with tools to manage their own disease. In contrast, only a few centres reported that they offered a written treatment plan for patients with COPD (Table 2). Other important aspects of tailoring the healthcare was to be available, supportive and “proactive” to prevent worsening.

*What I want is to… be available – I want to provide help, on the right level at the right time. What else do I want? Well, I want to be proactive /…/ I can maybe suggest that we initiate a treatment much earlier than many others would, or maybe just a little earlier than the recommendations say. (Professional 7)*

Interventions were tailored based on the patients’ needs, expectations and life situation. Non-smoking patients with a stable disease were given the responsibility to call whenever the need arose, while patients with recurrent exacerbations and smokers were prioritised for regular visits. Follow up of prioritised patients was offered by slightly more than half of the centres, while most centres offered treatment of exacerbations and had equipment (pulse oximeter, oxygen and nebuliser) for the purpose (Table 2).

Since it might take time for the patients to gain the insight they needed, the professionals expressed that they had to “go on instinct” to avoid to impose guilt and to get these shameful patients on board:

*…there’s so much talk about smoking that it ends up having a negative effect. People feel ashamed and /…/ don’t even want to go to the doctor because they can’t bear the idea of having to sit and listen to the sermon about smoking again. (Professional 8)*

### The autonomous staff

This category reflects how the weak healthcare organisation – for good or bad – has given room for autonomous and independent professionals to control their own work. The work with COPD is dependent on driving spirits and the attitudes of the professionals. Pragmatic work with routines may be a consequence of the lack of involvement from the management.

### Individual and shared responsibility

A shared responsibility within the profession was described for the everyday work with patients. For some this was an individual responsibility since they were the only representative of their profession in the centre. Management had a more general control at the centres, with no direct influence on the practical work:

*We just tell them ‘Now we’re going to do such-and-such.’ And they say ‘Oh, that’s great!’ And then we do it. (Professional 5)*

As long as the professionals could cope with the task without anyone else suffering they freely “ruled” over their own working methods and schedule. Sometimes the roles of the professionals were a bit “fuzzy at the edges” and a more pragmatic way of work where they helped each other was described.

Knowledge, experience and feeling of security were thought of as guiding the healthcare to patients with COPD and prioritisation of the work. For example, quality registers and assessment of patient-related outcomes were used only when an obvious advantage in their work was seen. A majority of the centres reported that they did not use the symptom assessment tools CAT (Chronic obstructive pulmonary disease Assessment Test) or mMRC (modified Medical Research Council scale), nor 6-min walk test to assess physical capacity (Table 2).

### Resistance to top-down directives and hierarchies

Resistance to some directives from the county council was expressed. The professionals tried not to be guided by financial incentives in clinical practice, unless it was an advantage in their work or directives compulsory for the primary care centres. Some were strongly critical to how economic incentives in primary care were negatively affected by activities (e.g. antibiotics prescription) in specialty care. Being monitored was still perceived as important, even though they felt frustrated since the theoretical image of civil servants and politicians sometimes was perceived inconsistent with reality:

*This is actually the ‘New Public Management’, if you’re familiar with the concept – that civil servants and politicians are supposed to control and impose a lot… (Professional 4)*

Discontent with hierarchies between professions was mentioned and physicians were occasionally described as a “complicated” professional group to work with. There were some experience of physicians trying to protect their position at the top of the hierarchy, to keep on being self-determining and to have power.

### Systematic versus pragmatic care development

A systematic versus a pragmatic – or even lacking – work with developing, implementing and following up routines were presented. National guidelines and locally designed routines was seen as important tools, although deficiencies were pointed out, such as no clear routines for screening patients for weight loss. A majority of the centres reported that they had a care programme for COPD care (Table 2). Systematic work with routines included regular, formal meetings where local routines were developed, discussed and documented based on guidelines. However, not all professionals were involved due to lack of time. The development of routines was also influenced by professional hierarchies. The body of physicians were by some described as having a strong professional identity and as occasionally having a clear dominance in care development, since it was difficult to go against their opinions.

The implementation of new routines was done progressively, in a kind of “trial and error” manner. Each individual was perceived as having the responsibility to implement the current routines in their work, with the result that not all professionals followed the routines. To secure the quality of their work, the professionals had a wish to follow up the effect of interventions and the satisfaction of the patients. Incident reporting was mentioned as one method they used, but even here keeping track of compliance to routines was the individual’s responsibility. An intuitively approach to follow up routines was more commonly formulated as: “it’s more that you notice that something’s not working”, which describes a more pragmatic approach to care development. The professionals also had an open dialogue at coffee and lunch breaks:

*But here we solve problems in the corridors. Decisions are made pretty quickly… ‘Yeah, okay, let’s do it this way’… and so that’s that and… You don’t have to go to a meeting at a particular time to discuss the issued, so it’s so much more convenient. (Professional 5)*

When working at a rather small centre this flexible way was seen as easy, however, a more structured way of working could also be advantageous.

### Driving spirit carries COPD care

The COPD nurse was defined as “a spider in the web” – a dedicated person with a key position, which coordinated the COPD care. The COPD nurse carried out many parts of the examination and treatment, such as performing spirometry and providing diagnosis, arranging referrals, educating the patients and providing medication, support for tobacco cessation and psychosocial support. If a patient still needed a physician contact, the COPD nurse could guide the patient to a physician who had shown more interest in and knowledge of the diagnosis. The other professions expressed a sense of security in knowing that the COPD nurse took responsibility for COPD, had the knowledge needed and was available to consult about COPD.

*Because as a doctor you have a lot of diagnoses, a lot of patients – quite a load then… it’s nice to be able to pass it off onto someone who can act as the administrative hub for all the services being provided to this patient. (Professional 2)*

Overall, this “administrative hub” provided some stability to the COPD care on an otherwise shaky organisational ground.

## Discussion

This is, to our knowledge, the first study using mixed methods to increase the understanding of what shapes the provision of healthcare services to people with COPD, and what healthcare is offered to people with COPD. The main results are represented by the theme *Building COPD care on shaky ground*. It symbolises the challenging circumstances and sometimes unfavourable work conditions in which the driven and independent primary care professionals work to provide supportive and empowering healthcare for patients with a complex disease, resulting in a less than ideal healthcare.

Confirming the subcategory *Shameful and low status disease*, patients with COPD have been reported to have less self-compassion and pride and more shame than healthy controls [43]. In a recent study [44] patients with COPD hospitalised with an exacerbation declined participation in PR since they felt guilt for causing the disease and did not consider themselves worthy of the healthcare offered. The low status in our result is reflected in the professionals’ perceived lack of competence, which could explain the low usage of assessment tools, along with the experience of health promotion as demanding. In a study of Swedish healthcare [45], the concept of health promotion was perceived as diffuse and hard to understand. At the same time Meis, et al. [46] pointed out the important role the professionals have when guiding the patients through lifestyle changes. In a study of implementation of multimodal pain rehabilitation – another low-status area in primary care in Sweden – it was pointed out that the management must take the responsibility to provide support and time for rehabilitation [47]. However, since the management in our study did not agree that patients with COPD was a group with low priority this might be a continuing problem.

Our study showed that professions like physiotherapists, occupational therapists, dieticians and medical social workers rarely were involved in COPD care at all. Meanwhile, earlier Swedish surveys [23, 24, 48] have reported high availability of professionals for rehabilitation in primary care. Access to patient education in groups was low in our study, while earlier studies [23, 24] showed that nearly half of the centres had access to PR-programmes in primary care, while a quarter sent their patients to hospital-based PR-programs [23]. As in our study, Cochrane, et al. [49] mentioned resources as one of several problems when implementing an interprofessional PR-programme. Furthermore, scepticism and lack of knowledge about how other professions work, along with hierarchies between professions, have also been confirmed by others [50, 51] as challenges for interprofessional collaboration. Even though hierarchies were described in our study, Sweden is considered a relatively non-hierarchical country where primary care centres are fairly autonomous. Overall, interprofessional collaboration is clearly limited and there are several challenges for the professionals and the management to deal with to improve the conditions for starting up interprofessional PR-programs.

The COPD care explored in our study is vulnerable and relies substantially on a COPD nurse with own personal motivation and drive, also described by Cochrane, et al. [49]. It could be interpreted as if the COPD care is hanging by a thread due to the lack of interprofessional collaboration. Furthermore, the COPD nurses in our study did not have sufficient supplementary training and time for COPD. According to national criteria for asthma/COPD receptions in primary care [39], a level corresponding to 10 weeks of university supplementary training about asthma and COPD for a COPD nurse is an acceptable level. Two third of the nurses in our study did not reach even half that level and similar results were found in a study covering the whole Sweden [48]. In the subcategory *Driving spirit carries COPD care* the COPD nurses was described to be main responsible for the spirometry tests, that is required to confirm a COPD diagnosis [1]. However, it is obvious that prevailing routines and time for diagnosing is not enough, which could contribute to the low prevalence for COPD in our study – only one tenth of the estimated national prevalence of 6.3% [4]. For optimal asthma/COPD receptions in primary care 4 h/week per 1000 enrolled citizens is recommended [39], while the COPD nurses in our study worked with COPD and asthma in median 1.4 h/week per 1000 enrolled citizens, which corresponds to an earlier Swedish study [48]. It has been shown that if more time is used, numbers of spirometries, pulse oximetries and weight measures increase, while reversibility tests and documentation of smoking habits decrease [52]. In addition, the patients have been shown to have fewer exacerbations per year at primary care centres with COPD nurses [48]. The professionals in our study pointed out that the COPD nurses were a good investment. This is confirmed in a study where overall direct cost savings were made when a specialty COPD nurse worked in inpatient care with spirometry, smoking cessation, lifestyle matters and follow up of acute exacerbation [53]. Consequently, the COPD nurses have a crucial role in COPD care. However, to prevent that COPD care is hanging by a thread, there is a need to expand it with interprofessional collaboration. In our study, there seem to be a considerable gap between the clinical practice and treatment guidelines for COPD. According to the national guidelines [40] people with COPD should be offered tailored healthcare with interprofessional collaboration. It should consist of education and support for self-care, such as a written treatment plan, and health promotion interventions focused on smoking cessation, exercise training and nutritional care. Furthermore, people with COPD should also be offered regular and structured follow ups and assessment of physical capacity and health status [40].

### Implications

This exploratory study is part of a larger project aiming to increase the availability to health promotion interventions for people with COPD in primary care using eHealth. Our study provides important information about conditions for implementation of such services and the findings may be translated to other similar settings. Based on the implementation framework i-PARIHS [25], the findings includes several contextual barriers for implementation of evidence based practice – the ‘shaky ground’ – such as low prioritisation for COPD, lack of interprofessional collaboration, limited support from management, and lacking resources. Furthermore, the health professionals, considered to be the recipients in i-PARISH [25], to a large extent lacked knowledge which also is a barrier for implementation. Consequently, the findings in the present study could inform the future development of strategies for implementation of health promotion interventions for people with COPD in the primary care. Such strategies need to target the identified barriers but also utilize the identified facilitators, such as the engagement of the COPD-nurses [25]. The Swedish National Board of Health and Welfare [40] have reported several interventions that is needed when implementing the guidelines; further training of professionals, increased availability to professionals, more time and more care contacts, and finally to create a functional structure for collaboration and patient education. These suggested interventions are also in line with the findings in our study. Already, a lot has happened in the county council since the data collection for this study, for example further training for spirometry has been given extra focus. However, a comprehensive effort is still needed to fully implement the national guidelines into clinical practice.

### Strengths and limitation

A major strength in our study was the use of mixed methods design. In an official recommendation of future research questions about COPD there is a distinct focus on quantitative research [54]. The qualitative component in this study contributed to an enriched answer of the research question. When the qualitative core component was complemented with descriptions from the quantitative supplemental component the results could give a more comprehensive picture [32], and this increase the strengths and decrease the weaknesses of each component included [31]. A limitation in this study is the general lack of patient perspective on perceptions such as the shameful patients.

During the process of data collection, analysis and description of this study, we have strived for trustworthiness [36] in several ways. First, in the process of ensuring credibility [36], we strived for a maximum variation sample. Partly the same sample was used for interviews and the questionnaire “Conceptual knowledge use”, to be able to compare the data from the same participants. However, sending the questionnaire to professionals at other centres as well could have gained the study, giving an opportunity to further broaden the knowledge of the professionals’ attitudes and intensions in the county council. Triangulation was performed between researchers with various competence and sex/gender, various methodological backgrounds and subjects, and with insider and outsider perspective, which is a strength of this study.

When this study was conducted, a discussion of the healthcare for people with COPD had started at a higher organisational level in the county council, since the first version of the new national, evidence based guidelines was released 2 months prior to our data collection. However, we strived for dependability [36] by performing the interviews during a short period of time, while the data collection for the questionnaires needed to be extended to ensure adequate response rate. To obtain transferability [36] we have presented a thorough description of the methods used and justifications why, as recommended for mixed methods [42] and in standards for reporting qualitative research [38]. Local autonomy within the healthcare system is a longstanding tradition in Sweden. However, the Swedish government has made efforts to impose more standardised healthcare practice across municipalities and county councils with national guidelines, national quality registers and open comparisons. The Västerbotten County differs slightly from Sweden as a whole with respect to demography and the existence of community hospitals with inpatient facilities in some of the most rural municipalities. Though, the effort to standardise the healthcare might have reduced the differences between the healthcare provided by the Swedish county councils. Since a gap between evidence and clinical practice is not only present in Sweden [18–20], our results could support future efforts to narrow the gap between evidence and practice in other settings with similar organisation and conditions.

### Further research

To further explore crucial and sometimes sensitive (professional and organisational) issues, such as how patterns of hierarchies and professions positions in the organisation influence the healthcare delivered to patients with COPD, more centres from different counties and international context, are desirable in future studies. Moreover, future studies are needed to explore the perspective of people with COPD, what healthcare they perceive is offered to them, what healthcare they accept and what affects their decision.

## Conclusions

This study implicates that the healthcare professionals are *Building COPD care on shaky ground* in primary care. They have the best intention for giving high quality care to this group of patients that are often uninformed and surrounded with shame, and it is the COPD nurse – a driving spirit – that is coordinating the COPD care. People with COPD are somewhat forgotten in a shaky organisation where the responsibility for COPD is fragmentised and has low priority. This study emphasises that there is a gap between evidence in the shape of treatment guidelines and the healthcare that is actually available to people with COPD. To be able to implement the guidelines in clinical practice several actions are needed, such as further training for the professionals, additional resources, and organisational improvements to increase interprofessional collaboration and patient education. There is also a need for future studies that explore the healthcare delivered to people with COPD from additional perspectives.

## Additional files

Additional file 1: Interview guide. Interview guide used for the semi-structured interviews. (PDF 67 kb)

Additional file 2: Questionnaire “Mapping of resources and interventions”*.* Questionnaire that was sent to senior managers at primary care centres in the first quantitative sample. (PDF 127 kb)

Additional file 3: Questionnaire “Conceptual knowledge use”. Questionnaire that was sent to healthcare professionals at two primary care centres in the second quantitative sample. (PDF 53 kb)

## Abbreviations

CAT: Chronic Obstructive Pulmonary Disease Assessment Test
COPD: Chronic Obstructive Pulmonary Disease
i-PARIHS: Integrated-Promoting Action on Research Implementation of Health Services
mMRC: Modified Medical Research Council scale
PARIHS: Promoting Action on Research Implementation of Health Services
PR: Pulmonary rehabilitation
SD: Standard deviation
