# Supplementary material for: Building COPD care on shaky ground: a mixed methods study from Swedish primary care professional perspective
Source: BMC Health Serv Res. 2017 Jul 10;17:467. doi: 10.1186/s12913-017-2393-y (PMC5504776; doi:10.1186/s12913-017-2393-y)
Supplement: Supplementary file 1 — Interview guide. Interview guide used for the semi-structured interviews. (PDF 67 kb) [file 12913_2017_2393_MOESM1_ESM.pdf]

Interview guide

What is your experience of care and treatment for people with COPD?

How do you work with people with COPD? What do you want to achieve?

What do you think affects how care and interventions for people with COPD are shaped in your organisation/at your unit?

How do you create new work routines for how treatment and care are designed/carried out in your organisation/at your unit?

How does the use of guidelines work in practice at your workplace?

Describe your confidence in working according to national guidelines.

How are care and treatment followed up in your organisation/at your unit?

Are there areas within the care provided to people with COPD where there is disagreement, i.e. were care providers have different ideas about what is optimal care? What are these and how are they handled?

Is there anything you would like to change regarding care and initiatives for people with COPD?

What further training and/or supervision in COPD rehabilitation exist at your workplace?

Tell us what other strategies you have to learn things you need in your work – and how effective you think these strategies are?
