# Supplementary material for: Building COPD care on shaky ground: a mixed methods study from Swedish primary care professional perspective
Source: BMC Health Serv Res. 2017 Jul 10;17:467. doi: 10.1186/s12913-017-2393-y (PMC5504776; doi:10.1186/s12913-017-2393-y)
Supplement: Supplementary file 2 — Questionnaire “Mapping of resources and interventions”. Questionnaire that was sent to senior managers at primary care centres in the first quantitative sample. (PDF 127 kb) [file 12913_2017_2393_MOESM2_ESM.pdf]

Survey of access to healthcare in the primary care sector for people with chronic obstructive pulmonary disease (COPD)

1. Enter the name of the primary care centre.

____

2. How many patients are enrolled at the primary care centre?

____

3. How many of the patients enrolled at the primary care centre have been diagnosed with COPD (diagnosis code J44)?

____

4. Does the primary care centre have a physician medically responsible specifically for patients with asthma and COPD?

☐ Yes
☐ No

5. Is there an asthma/COPD nurse at the primary care centre?
Asthma/COPD nurse refers to a nurse specifically tasked with responsibility for patients diagnosed with asthma and COPD. If there is other staff with a specialised function in the area of asthma and COPD at the primary care centre, please describe this at "Other".

☐ Yes
☐ No
☐ Other: ____

6. What supplementary training does the asthma/COPD nurse have?
If you chose "Other" in the preceding question, please specify the supplementary training of that person.

☐ No advanced training in the area of asthma/COPD
☐ 7.5 credits in the area of asthma/COPD
☐ 15 credits in the area of asthma/COPD
☐ 30 credits in the area of asthma/COPD
☐ Other education/training, please specify: ____

7. How many hours of the asthma/COPD nurse's working time each week are designated for work with asthma patients?
If the asthma/COPD nurse also works with general smoking cessation support, i.e. smoking cessation support not targeting individuals with asthma, then these hours should not be included.

____

8. How many hours of the asthma/COPD nurse's working time each week are designated for work with COPD patients?
If the asthma/COPD nurse also works with general smoking cessation support, i.e. smoking cessation support not targeting individuals with COPD, then these hours should not be included.

____

9. How many actual hours of the asthma/COPD nurse's working time each week are used for work with asthma patients?

____

10. How many actual hours of the asthma/COPD nurse's working time each week are used for work with COPD patients?

____

11. How many full-time positions of the professional categories below are found at the primary care centre?
Choose the alternative that is closest.

	0	0.1-0.5	0.6-0.9	1	2	3	4	> 4
Physician	☐	☐	☐	☐	☐	☐	☐	☐
Nurse/district nurse (including asthma/COPD nurse)	☐	☐	☐	☐	☐	☐	☐	☐
Assistant nurse	☐	☐	☐	☐	☐	☐	☐	☐
Physiotherapist	☐	☐	☐	☐	☐	☐	☐	☐
Occupational therapist	☐	☐	☐	☐	☐	☐	☐	☐
Dietitian	☐	☐	☐	☐	☐	☐	☐	☐
Medical social worker	☐	☐	☐	☐	☐	☐	☐	☐
Psychologist	☐	☐	☐	☐	☐	☐	☐	☐

12. Which staff category/categories work with patients with COPD?
☐ Physician
☐ Nurse/district nurse (not including the asthma/COPD nurse)
☐ Assistant nurse
☐ Physiotherapist
☐ Occupational therapist
☐ Dietitian
☐ Medical social worker
☐ Psychologist

☐ Other, please specify: ____

13. How many working hours per week are designated for working with patients with COPD for the different professional categories?
If multiple people in the same professional category have time designated for working with patients with COPD, add together their hours.

	0	1-2	3-4	5-8	9-12	13-16	17-20	21-24	25-28	29-32	33-36	37-40	> 40
Physician	☐	☐	☐	☐	☐	☐	☐	☐	☐	☐	☐	☐	☐
Nurse/district nurse (including asthma/COPD nurse)	☐	☐	☐	☐	☐	☐	☐	☐	☐	☐	☐	☐	☐
Assistant nurse	☐	☐	☐	☐	☐	☐	☐	☐	☐	☐	☐	☐	☐
Physiotherapist	☐	☐	☐	☐	☐	☐	☐	☐	☐	☐	☐	☐	☐
Occupational therapist	☐	☐	☐	☐	☐	☐	☐	☐	☐	☐	☐	☐	☐
Dietitian	☐	☐	☐	☐	☐	☐	☐	☐	☐	☐	☐	☐	☐
Medical social worker	☐	☐	☐	☐	☐	☐	☐	☐	☐	☐	☐	☐	☐
Psychologist	☐	☐	☐	☐	☐	☐	☐	☐	☐	☐	☐	☐	☐

14. How many actual working hours per week are used for working with patients with COPD for the different professional categories?
If multiple people in the same professional category have time designated for working with patients with COPD, add together their hours.

	0	1-2	3-4	5-8	9-12	13-16	17-20	21-24	25-28	29-32	33-36	37-40	> 40
Physician	☐	☐	☐	☐	☐	☐	☐	☐	☐	☐	☐	☐	☐
Nurse/district nurse (including asthma/COPD nurse)	☐	☐	☐	☐	☐	☐	☐	☐	☐	☐	☐	☐	☐
Assistant nurse	☐	☐	☐	☐	☐	☐	☐	☐	☐	☐	☐	☐	☐
Physiotherapist	☐	☐	☐	☐	☐	☐	☐	☐	☐	☐	☐	☐	☐
Occupational therapist	☐	☐	☐	☐	☐	☐	☐	☐	☐	☐	☐	☐	☐
Dietitian	☐	☐	☐	☐	☐	☐	☐	☐	☐	☐	☐	☐	☐
Medical social worker	☐	☐	☐	☐	☐	☐	☐	☐	☐	☐	☐	☐	☐
Psychologist	☐	☐	☐	☐	☐	☐	☐	☐	☐	☐	☐	☐	☐

15. Is there any further/supplementary training in COPD available to the staff?

☐ Yes
☐ No

16. Please provide a brief description of the COPD further/supplementary training available.

____

____

17. In relation to interventions for patients with COPD, is a defined care programme applied at the primary care centre?

☐ Yes
☐ No

18. Who developed the care programme for COPD?

____

19. Which of the following measures does the primary care centre offer to patients with COPD?
- ☐ Spirometry
- ☐ Structured investigations with spirometry and reversibility testing
- ☐ Treatment of exacerbations
- ☐ Follow-up of prioritised patients in accordance with the National Board of Health and Welfare's guidelines
- ☐ Symptom assessment with CAT
- ☐ Symptom assessment with mMRC
- ☐ Patient education (in addition to individual consultation)
- ☐ Written treatment plan
- ☐ Smoking cessation support
- ☐ Designated time for patient counselling with the asthma/COPD nurse in person or by phone
- ☐ 6-minute walk test
- ☐ Physical exercise/activity as part of the treatment (with physiotherapist)
- ☐ Consultative discussions about physical exercise/activity
- ☐ Physical activity on prescription (FaR)
- ☐ Consultative discussions about diet and energy needs
- ☐ Consultative discussions about energy-saving measures and assistive devices
- ☐ Instruction and follow-up of inhalation techniques
- ☐ Other, please specify: ____

20. Please provide a brief description of the structure and content in the patient education.

____

____

21. Please indicate which professional categories are involved in the patient education.
- ☐ Physician
- ☐ Asthma/COPD nurse
- ☐ Nurse/district nurse
- ☐ Assistant nurse
- ☐ Physiotherapist
- ☐ Occupational therapist
- ☐ Dietitian
- ☐ Medical social worker
- ☐ Psychologist
- ☐ Other, please specify: ____

22. Which of the following equipment is available at the primary care centre?
   - ☐ Spirometer
   - ☐ Pulse oximeter
   - ☐ Oxygen
   - ☐ Nebuliser

23. Are there routines at the primary care centre for asking patients about their tobacco habits?
   - ☐ Yes
   - ☐ No

24. Please provide a brief description of the routines for asking patients about their tobacco habits.

    ____

    ____

25. Does the primary care centre offer smoking cessation support?
    ☐ Yes
    ☐ No

26. What professional categories offer smoking cessation support?

    ____

27. What percentage of patients who are or were previously smokers are offered a spirometry examination?
    ☐ 0–25%
    ☐ 25–50%
    ☐ 50–75%
    ☐ 75–100%
    ☐ Not routinely offered

28. Research shows that health promotion interventions have positive effects on people with COPD, but only a small number have access to such interventions. Are people with COPD routinely offered health promotion interventions at the primary care centre?
    In this context, health promotion interventions refers to physical exercise/activity or consultative discussions about physical exercise/activity, information about self-care strategies and breathing techniques.
    ☐ Yes
    ☐ No
    ☐ Other, please specify: ____

29. What difficulties have you encountered when it comes to offering patients with COPD health promotion interventions (as described in question 28)?
    ☐ No resources for this purpose
    ☐ The patient group is not prioritised
    ☐ The patients are not interested in participating in health promotion interventions
    ☐ The patients feel that they do not have the energy to travel to the health centre to participate in health promotion interventions
    ☐ The staff do not have adequate knowledge to be able to provide health promotion interventions
    ☐ The staff are doubtful about the effect of health promotion interventions
    ☐ We have not encountered any difficulties
    ☐ Other, please specify: ____

30. Additional comments or viewpoints:
    We welcome any comments you may have about the questions or other viewpoints you have regarding interventions in primary care for patients with COPD.

    ____

    ____

31. May we contact you by phone if we have any questions about your responses to the survey or if you missed answering any question?
    ☐ No, I do not wish to be contacted
    ☐ Yes, you can contact me at the following phone number: ____________________________
