# Supplementary material for: Building COPD care on shaky ground: a mixed methods study from Swedish primary care professional perspective
Source: BMC Health Serv Res. 2017 Jul 10;17:467. doi: 10.1186/s12913-017-2393-y (PMC5504776; doi:10.1186/s12913-017-2393-y)
Supplement: Supplementary file 3 — Questionnaire “Conceptual knowledge use”. Questionnaire that was sent to healthcare professionals at two primary care centres in the second quantitative sample. (PDF 53 kb) [file 12913_2017_2393_MOESM3_ESM.pdf]

Best,

A number of questions are found below. We ask that you please answer them based on *your own perception/experience of* the subject matter of the question.

Thank you in advance for your participation.

Background information

Sex/gender:____________________________ Year of birth:________________________

Current position:____

Number of years in current position:_________

Number of years in the profession:__________

Supplementary/further training:____

How many patients with COPD do you meet during a regular working week?

☐	☐	☐	☐	☐	☐
0	1–2	3–5	6–10	11–15	> 15

1. Health promotion interventions

a. Offering health promotion interventions to people with COPD is an important task in primary care (please tick the box that best describes your opinion).

☐	☐	☐	☐
Disagree	Agree to some extent	Largely agree	Agree completely

b. Do you feel like you have sufficient knowledge to offer health promotion interventions to people with COPD?

☐ Yes, I essentially have sufficient knowledge

☐ No, I do not have sufficient knowledge

c. Do your work tasks include providing health promotion interventions to people with COPD?

☐ Yes

☐ No

d. *If your work tasks include* providing health promotion interventions to people with COPD, have you encountered any difficulties in providing these interventions/activities?

☐ Yes ☐ No

If yes, please describe ____

____

____

2. Tobacco prevention

a. Offering tobacco prevention to people with COPD is an important task in primary care (please tick the box that best describes your opinion).

☐	☐	☐	☐
Disagree	Agree to some extent	Largely agree	Agree completely

b. Do you feel like you have sufficient knowledge to offer tobacco prevention to people with COPD?

☐ Yes, I essentially have sufficient knowledge ☐ No, I do not have sufficient knowledge

c. Do your work tasks include providing tobacco prevention to people with COPD?

☐ Yes ☐ No

d. *If your work tasks include* providing tobacco prevention to people with COPD, have you encountered any difficulties in providing these interventions/activities?

☐ Yes ☐ No

If yes, please describe ____

____

____

3. Disease-specific instruction

a. Offering disease-specific education to people with COPD is an important task in primary care (please tick the box that best describes your opinion).

☐	☐	☐	☐
Disagree	Agree to some extent	Largely agree	Agree completely

b. Do you feel like you have sufficient knowledge to offer disease-specific education to people with COPD?

☐ Yes, I essentially have sufficient knowledge ☐ No, I do not have sufficient knowledge

c. Do your work tasks include providing disease-specific education to people with COPD?

☐ Yes ☐ No

d. *If your work tasks include* providing disease-specific education to people with COPD, have you encountered any difficulties in providing this intervention/activity?

☐ Yes ☐ No

If yes, please describe ____

____

____

4. Self-care strategies

a. Providing information on strategies for COPD-related self-care (e.g. information on for which symptoms an individual should seek healthcare) to people with COPD is an important task in primary care (please tick the box that best describes your opinion).

☐	☐	☐	☐
Disagree	Agree to some extent	Largely agree	Agree completely

b. Do you feel like you have sufficient knowledge to provide information on strategies for COPD-related self-care to people with COPD?

☐ Yes, I essentially have sufficient knowledge ☐ No, I do not have sufficient knowledge

c. Do your work tasks include providing information on strategies for COPD-related self-care to people with COPD?

☐ Yes ☐ No

d. *If your work tasks include* providing information on strategies for COPD-related self-care to people with COPD, have you encountered any difficulties in providing this intervention/activity?

☐ Yes ☐ No

If yes, please describe ____

____

____

5. Physical activity/exercise training

a. Offering consultative discussions about physical activity/exercise training to people with COPD is an important task in primary care (please tick the box that best describes your opinion).

☐	☐	☐	☐
Disagree	Agree to some extent	Largely agree	Agree completely

b. Do you feel like you have sufficient knowledge to provide consultative discussions about physical activity/exercise training to people with COPD?

☐ Yes, I essentially have sufficient knowledge ☐ No, I do not have sufficient knowledge

c. Do your work tasks include providing consultative discussions about physical activity/exercise training to people with COPD?

☐ Yes ☐ No

d. *If your work tasks include* providing consultative discussions about physical activity/exercise training to people with COPD, have you encountered any difficulties in providing this intervention/activity?

☐ Yes ☐ No

If yes, please describe ____

____

____

6. Physical activity on prescription

a. Offering physical activity on prescription to people with COPD is an important task in primary care (please tick the box that best describes your opinion).

☐	☐	☐	☐
Disagree	Agree to some extent	Largely agree	Agree completely

b. Do you feel like you have sufficient knowledge to offer physical activity on prescription to people with COPD?

☐ Yes, I essentially have sufficient knowledge   ☐ No, I do not have sufficient knowledge

c. Do your work tasks include providing physical activity on prescription to people with COPD?

☐ Yes   ☐ No

d. *If your work tasks include* providing physical activity on prescription to people with COPD, have you encountered any difficulties in providing this intervention/activity?

☐ Yes   ☐ No

If yes, please describe ____

____

____

7. Breathing techniques

a. Offering instruction and training in breathing techniques to people with COPD is an important task in primary care (please tick the box that best describes your opinion).

☐	☐	☐	☐
Disagree	Agree to some extent	Largely agree	Agree completely

b. Do you feel like you have sufficient knowledge to provide instruction and training in breathing techniques to people with COPD?

☐ Yes, I essentially have sufficient knowledge   ☐ No, I do not have sufficient knowledge

c. Do your work tasks include providing instruction and training in breathing techniques to people with COPD?

☐ Yes   ☐ No

d. *If your work tasks include* providing instruction and training in breathing techniques to people with COPD, have you encountered any difficulties in providing this intervention/activity?

☐ Yes ☐ No

If yes, please describe ____

____

____

8. Nutrition and energy needs

a. Offering consultative discussions about nutrition and energy needs to people with COPD is an important task in primary care (please tick the box that best describes your opinion).

☐	☐	☐	☐
Disagree	Agree to some extent	Largely agree	Agree completely

b. Do you feel like you have sufficient knowledge to provide consultative discussions about nutrition and energy needs to people with COPD?

☐ Yes, I essentially have sufficient knowledge ☐ No, I do not have sufficient knowledge

c. Do your work tasks include providing consultative discussions about nutrition and energy needs to people with COPD?

☐ Yes ☐ No

d. *If your work tasks include* providing consultative discussions about nutrition and energy needs to people with COPD, have you encountered any difficulties in providing this intervention/activity?

☐ Yes ☐ No

If yes, please describe ____

____

____

9. Energy conservation techniques and assistive devices

a. Providing information on energy conservation techniques and assistive devices to people with COPD is an important task in primary care (please tick the box that best describes your opinion).

☐	☐	☐	☐
Disagree	Agree to some extent	Largely agree	Agree completely

b. Do you feel like you have sufficient knowledge to provide information on energy conservation techniques and assistive devices to people with COPD?

☐ Yes, I essentially have sufficient knowledge

☐ No, I do not have sufficient knowledge

c. Do your work tasks include providing information on energy conservation techniques and assistive devices to people with COPD?

☐ Yes

☐ No

d. *If your work tasks include* providing information on energy conservation techniques s and assistive devices to people with COPD, have you encountered any difficulties in providing this intervention/activity?

☐ Yes

☐ No

If yes, please describe ____

____

____

10. Other interventions/activities

a. Are there any interventions/activities not mentioned in this survey that you would like to offer people with COPD?

☐ Yes

☐ No

If yes, please describe____

____

____

b. If you entered any interventions/activities at question 10a, have you experienced any difficulties in providing these interventions/activities to people with COPD?

☐ Yes

☐ No

If yes, please describe ____

____

____
